# Supplementary material for: A mechanistic insight into sources of error of visual working memory in multiple sclerosis
Source: eLife. 2023 Nov 8;12:RP87442. doi: 10.7554/eLife.87442 (PMC10631758; doi:10.7554/eLife.87442)
Supplement: Supplementary file 1. [file elife-87442-supp1.docx]

**Table 1. Hierarchical regression analysis for the MGL paradigm.**

|  | **Statistical reports** |
| --- | --- |
| **Independent variables** | **Recall parameters**  **Error/ Precision (distance)/ Precision (delay) (*F*, *P*)** |
| **Group** | (14.57, < 10^-5^*)/ (13.74, <10^-4^*)/ (14.57, < 10^-5^*) |
| **Group**  **Cognitive ability** | (10.91, < 10^-4^*/ (9.7, < 10^-3^*)/ (10.62, < 10^-3^*)  (24.01, < 10^-5^*)/ (6.29, <0.02*)/ (5.59, < 0.03*) |

Cognitive ability: assessed based on the Montreal cognitive assessment (MoCA) test classification.

MGL = Memory-guided localization

****P* < 0.05**
