# Supplementary material for: A mechanistic insight into sources of error of visual working memory in multiple sclerosis
Source: eLife. 2023 Nov 8;12:RP87442. doi: 10.7554/eLife.87442 (PMC10631758; doi:10.7554/eLife.87442)
Supplement: Supplementary file 2. [file elife-87442-supp2.docx]

**Table 1. Statistical results of reaction time (in second) for the MGL paradigm.**

|  | **Statistical results** |
| --- | --- |
| Healthy | 1.59 ± 0.41 |
| RRMS | 2.33 ± 0.65 |
| SPMS | 3.02 ± 0.87 |
| Between groups (mixed-model ANOVA) | *F*(2,61) = 26.44, *P* < 10^-8^* |
| Healthy vs. RRMS (Tukey) | *P* < 0.004* |
| Healthy vs. SPMS (Tukey) | *P* < 10^-8^* |
| RRMS vs. SPMS (Tukey) | *P* < 0.008* |
| Within group (Distance, mixed-model ANOVA) | *F*(2,61) = 25.94, *P* < 10^-9^* |
| Interaction (Group × Distance, mixed-model ANOVA) | *F*(4,61) = 2.06, *P* = 0.09 |
| Within group (Delay, mixed-model ANOVA) | *F*(4,61) = 0.97, *P* = 0.43 |
| Interaction (Group × Delay, mixed-model ANOVA) | *F*(8,61) = 0.86, *P* = 0.55 |

MGL = Memory-guided localization, RRMS = Relapsing-remitting multiple sclerosis, SPMS = Secondary progressive multiple sclerosis.

Data are represented as mean ± standard deviation.

****P* < 0.05**
