# Supplementary material for: A mechanistic insight into sources of error of visual working memory in multiple sclerosis
Source: eLife. 2023 Nov 8;12:RP87442. doi: 10.7554/eLife.87442 (PMC10631758; doi:10.7554/eLife.87442)
Supplement: Supplementary file 3. [file elife-87442-supp3.docx]

**Table 1. Hierarchical regression analysis for the sequential paradigm with 3 bar, high memory load condition.**

|  | **Statistical reports** |
| --- | --- |
| **Independent variables** | **Recall parameters**  **Error/ Precision/ von Mises SD/ Target proportion/ Swap error/ Uniform proportion / Nearest neighbor (*F*, *P*)** |
| **Group** | (28.18, < 10^-9^*) / (25.23, < 10^-9^*)/ (26.79, < 10^-9^*)/ (11.04, < 10^-4^*)/ (7.11, < 0.002*)/ (5.50, < 0.006*)/ (29.26, < 10^-10^*) |
| **Group**  **Gender** | (22.47, < 10^-8^*)/ (19.90, < 10^-7^*)/ (20.37, < 10^-7^*)/ (9.98, < 0.0002*)/ (7.22, < 0.002*)/ (4.38, < 0.02*)/ (22.64, < 10^-8^*)  (3.89, = 0.051)/ (4.16, < 0.05*)/ (8.71, < 0.004*)/ (0.002, = 0.97)/ (0.47, = 0.49)/ (0.57, = 0.45)/ (7.82, < 0.007*) |
| **Group**  **Age** | (18.84, < 10^-7^*)/ (17.68, < 10^-6^*)/ (18.53, < 10^-6^*)/ (6.76, < 0.002*)/ (6.32, < 0.003*)/ (2.85, = 0.06)/ (19.60, < 10^-7^*)  (9.39, < 0.003*)/ (7.80, < 0.007*)/ (5.51, < 0.03*)/ (5.24, < 0.03*)/ (0.07, = 0.79)/ (15.04, < 0.0002*)/ (12.04, < 10^-3^*) |
| **Group**  **Education** | (14.03, < 10^-5^*)/ (12.79, < 10^-4^*)/ (15.22, < 10^-5^*)/ (4.17, < 0.02*)/ (4.33, < 0.02*)/ (0.90, = 0.41)/ (14.2, < 10^-5^*)  (8.21, < 0.005*)/ (5.46, < 0.03*)/ (3.21, = 0.08)/ (6.64, < 0.02*)/ (0.52, = 0.47)/ (10.08, < 0.002*)/ (9.57, 0.003*) |
| **Group**  **Cognitive ability^a^** | (19.26, < 10^-7^*)/ (18.23, < 10^-6^*)/ (19.69, < 10^-7^*)/ (5.71, < 0.005*)/ (3.41, < 0.04*)/ (2.95, = 0.056)/ (20.93, < 10^-7^*)  (14.91, < 10^-3^*)/ (6.81, < 0.02*)/ (5.68, 0.02*)/ (16.10 < 10^-3^*)/ (11.82, < 10^-3^*)/ (5.73, < 0.02*)/ (8.94, < 0.004*) |

Cognitive ability: assessed based on the Montreal cognitive assessment (MoCA) test classification.

^a^ One MoCA value in SPMS group is missing.

****P* < 0.05**
