# Supplementary material for: A mechanistic insight into sources of error of visual working memory in multiple sclerosis
Source: eLife. 2023 Nov 8;12:RP87442. doi: 10.7554/eLife.87442 (PMC10631758; doi:10.7554/eLife.87442)
Supplement: Supplementary file 4. [file elife-87442-supp4.docx]

**Table 1. Statistical results of reaction time (in second) for the sequential paradigms (3-bar and 1-bar)**

|  | **Statistical results (3-bar)** | **Statistical results (1-bar)** |
| --- | --- | --- |
| Healthy | 2.62 ± 1.01 | 2.25 ± 0.92 |
| RRMS | 3.14 ± 0.96 | 2.78 ± 1.04 |
| SPMS | 4.18 ± 2.00 | 3.66 ± 1.72 |
| Between groups (mixed-model ANOVA) | *F*(2,114) = 12.95, *P* < 10^-5^* | *F*(2,114) = 12.59, *P* < 10^-4^* |
| Healthy vs. RRMS (Tukey) | *P* = 0.17 | *P* = 0.11 |
| Healthy vs. SPMS (Tukey) | *P* <10^-5^* | *P* < 10^-5^* |
| RRMS vs. SPMS (Tukey) | *P* < 0.005* | *P* < 0.009* |
| Within group (Bar order, mixed-model ANOVA) | *F*(2,114) = 5.92, *P* < 0.004* | N/A |
| Interaction (Group × Bar order, mixed-model ANOVA) | *F*(4,114) = 0.16, *P* = 0.96 | N/A |

RRMS = Relapsing-remitting multiple sclerosis, SPMS = Secondary progressive multiple sclerosis.

Data are represented as mean ± standard deviation.

****P* < 0.05**
