# Supplementary material for: A mechanistic insight into sources of error of visual working memory in multiple sclerosis
Source: eLife. 2023 Nov 8;12:RP87442. doi: 10.7554/eLife.87442 (PMC10631758; doi:10.7554/eLife.87442)
Supplement: Supplementary file 5. [file elife-87442-supp5.docx]

**Table 1. Hierarchical regression analysis for the sequential paradigm with 1 bar, low memory load condition.**

|  | **Statistical reports** |
| --- | --- |
| **Independent variables** | **Recall parameters**  **Error/ Precision/ von Mises SD/ Target or Uniform proportion (*F*, *P*)** |
| **Type** | (36.85, < 10^-12^*)/ (25.48, < 10^-9^*)/ (33.07, < 10^-11^*)/ (3.11, < 0.05*) |
| **Group**  **Gender** | (29.46, < 10^-10^*)/ (20.05, < 10^-7^*)/ (26.92, < 10^-9^*)/ (1.97, = 0.14)  (8.09, < 0.006*)/ (4.41, < 0.04*)/ (3.45, = 0.07)/ (2.44, = 0.12) |
| **Group**  **Age** | (23.85, < 10^-8^*)/ (18.48, < 10^-6^*)/ (22.03, < 10^-8^*)/ (1.87, = 0.16)  (8.27, < 0.005*)/ (1.57, = 0.21)/ (6.89, < 0.001*)/ (0.57, = 0.45) |
| **Group**  **Education** | (23.04, < 10^-8^*)/ (15.41, < 10^-5^*)/ (18.65, < 10^-6^*)/ (2.59, = 0.08)  (4.03, < 0.05*)/ (2.42, = 0.12)/ (6.36, < 0.02*)/ (0.01, = 0.91) |
| **Group**  **Cognitive ability^a^** | (27.35, 10^-9^*)/ (20.6, < 10^-7^*)/ (25.54, < 10^-9^*)/ (1.87, = 0.16)  (4.46, < 0.04*)/ (0.35, = 0.56)/ (5.25, < 0.03*)/ (0.08, = 0.77) |

Cognitive ability: assessed based on the Montreal cognitive assessment (MoCA) test classification.

^a^ One MoCA value in SPMS group is missing.

****P* < 0.05**
